# Exploring Drug-Related Problems in Diabetic Patients during Ramadan Fasting in Saudi Arabia: A Mixed-Methods Study

**DOI:** 10.3390/ijerph16030499

**Published:** 2019-02-11

**Authors:** Waleed Saleh Alluqmani, Mohammed Misri Alotaibi, Waleed Jamal Almalki, Abdulrahman Althaqafi, Hamed Abdullah Alawi, Fahad Althobiani, Amjad Abdullah Albishi, Amjad Ahmad Madkhali, Layal Yahia Baunes, Razan Ibrahim Alhazmi, Elaf Mohammed Doman, Anwar Hussain Alhazmi, Majid Ali, Ejaz Cheema

**Affiliations:** 1 College of Pharmacy, Umm al-Qura University, Taif Road, Makkah 24381, Saudi Arabia; waleed9055@hotmail.com (W.S.A.); momsmf@hotmail.com (M.M.A.); ph.waleed.ja@gmail.com (W.J.A.); abdulrhmanthaqafi@gmail.com (A.A.); hamed234@windowslive.com (H.A.A.); fahaddakilallh@gmail.com (F.A.); ph-jad@hotmail.com (A.A.A.); ph.amjaad@hotmail.com (A.A.M.); laaayaly-2009@windowslive.com (L.Y.B.); razxhr@gmail.com (R.I.A.); e_m_h_d@hotmail.com (E.M.D.); alhazmi.anwar.h@gmail.com (A.H.A.); majid.ali@hotmail.com (M.A.); 2 Institute of Clinical Sciences, University of Birmingham, Edgbaston, Birmingham B15 2TT, UK

**Keywords:** medicines, diabetes, Ramadan, fasting

## Abstract

This study aimed to identify any drug-related problems (DRPs) in diabetic patients during Ramadan fasting in Saudi Arabia. The study used a mixed-methods approach consisting of two phases and was conducted in Makkah, Saudi Arabia from December 2017 to March 2018. The first phase of the study involved qualitative semi-structured individual interviews with diabetic patients. A 13-item questionnaire was used in the second phase to further identify DRPs in the wider population. The data was mainly presented as frequencies and percentages. Inferential statistics was performed using Statistical Package for Social Sciences (SPSS) version 21 to compare relevant variables/questions using the chi-square test. Twenty patients (10 male, 10 female) attended face-to-face interviews during the first phase of the study while 95 (40 male, 55 female) completed the questionnaire in the second phase of the study. Two possible risk factors for DRPs were identified from the qualitative data: patient-related factors, including changes in their medicine intake during fasting, and healthcare professionals-related factors, including lack of advice from healthcare professionals regarding fasting. The quantitative results indicated that 52 (54%) of the 95 participants who observed fasting reported to have changed the way they were taking their medicines. Furthermore, 41% of the participants experienced general healthcare problems such as hypoglycemia, hyperglycemia, fatigue, excessive sweating, and gastrointestinal disturbances. Healthcare professionals need to educate patients who are at risk of DRPs by providing structured education and counseling.

## 1. Introduction

Diabetes is a common chronic disease with a major social, health, and economic impact. It is estimated that in 2017, there were 425 million people (20–79 years of age) with diabetes worldwide and it is expected that this number would increase to 629 million by 2045 [1]. Saudi Arabia is ranked second in the Middle East and seventh in the world for the prevalence of diabetes with an estimated 7 million population suffering from diabetes [2].

Each year, Muslims all over the world including Saudi Arabia fast during the month of Ramadan which requires them to refrain from any food and drink including medicines from dawn to sunset [3]. Although patients with chronic diseases such as diabetes are not required to fast as per Islamic rules, many of them insist on fasting during Ramadan [4]. A multi-center study conducted in as many as 13 countries including Algeria, Bangladesh, Egypt, India, Indonesia, Jordan, Lebanon, Malaysia, Morocco, Pakistan, Saudi Arabia, Tunisia, and Turkey suggested that 78% of the diabetic population are expected to fast during the month of Ramadan [5]. Evidence suggests that may patients who fast may alter the daily dosage intake of their medicines without seeking medical advice from healthcare professionals [6]. The changes in the medication schedule coupled with the changes in eating habits in Ramadan can put the patients who fast to be at risk of drug-related problems (DRPs). Other factors that may contribute to the development of DRPs in diabetic patients include longer duration of fasting period particularly in certain Western countries and changes in lifestyle including lack of physical activity [5].

A DRP can be defined as an event or a circumstance involving drug therapy that actually or potentially interferes with the desired health outcome [7]. The Pharmaceutical Care Network Europe (PCNE) further categorizes the DRPs based on the causes of the problem that includes the selection of inappropriate drug therapy, medication errors, overuse or underuse of drugs, adverse drug reactions, drug-drug interactions, and drug-food interactions [7]. It is important for the healthcare professionals to be aware of the implications of potential DRPs on glycemic control of patients who may already be at risk of developing other complications including hypoglycemia, hyperglycemia, thrombosis and dehydration due to longer fasting periods [8,9,10]. Although there have been some studies that have explored diabetic patients’ perceptions and attitudes towards their medications and diet intake during Ramadan [10,11], no such study has been conducted in a largely Muslim-dominated country such as Saudi Arabia. This study, therefore, aims to explore diabetic patients’ practices towards their medications and diet intake during fasting and to identify any DRPs during Ramadan in Saudi Arabia.

## 2. Methods

### 2.1. Study Design

This study used a mixed-methods approach consisting of both qualitative and quantitative phases and was conducted in Makkah, Saudi Arabia from December 2017 to March 2018.

### 2.2. Ethical Approval

The study was approved by the Umm Al-Qura University Human Research Ethics Committee [UQU-COP-EA # 143623] and by Al-Noor Hospital Diabetic Center [431238]. A participant information sheet was provided to all participants and informed consent was obtained prior to the start of the study.

The research was conducted in two phases:

• Phase I—Qualitative 

This phase involved face-to-face semi-structured interviews with diabetic patients who were attending Diabetes Center of a large governmental hospital in Makkah, Saudi Arabia. Adults aged 18 years or over, male or female and capable of giving written consent were eligible for inclusion. Eligible patients were identified and approached by the members of the research team. Participants were recruited on their willingness and ability to participate. In other words, the sample population was a convenience sample of patients. An interview guide based on some previous studies [12,13,14,15] was developed in English and then translated into Arabic language (Appendix A: Interview guide). The interview guide was developed to help authors explore participant’s views about their management of diabetes during Ramadan including medicine intake, monitoring of blood glucose levels and experiences of DRPs. Furthermore, the interview guide explored participants’ lifestyle including eating habits and type of physical activities undertaken during Ramadan fasting. The interview guide was piloted on a small number of patients (*n* = 5) and based on their feedback, minor changes were made. These changes included rephrasing the language of two questions to make it easier for understanding. Eligible participants were approached by two members of the research team who were trained by a senior academic to conduct face-to-face interviews. Each interview was conducted individually that lasted around 15 min. All interviews were audio recorded with the consent of patients and transcribed verbatim. Qualitative data was analyzed using the inductive method of thematic analysis [16]. The initial step of the analysis involved familiarizing with the data through reading of the whole data to familiarize with all participants’ responses. It was followed by manual generation of initial codes from the data. The coding process was independently verified by a senior academic (M.A.) and any variations in the coding process were resolved through discussion. Potential themes were then assigned to the generated codes. Themes were reviewed to ensure they were appropriate with the exclusion of some of the initial themes due to the availability of limited data related to the excluded themes. The final themes were then defined and named by the members of research team.

• Phase II—Quantitative

Based on the findings of Phase I of the study, a 13-item questionnaire was prepared in the phase II to further identify DRPs in the wider population. The questionnaire was piloted on a small number of patients (*n* = 5). However, no changes were made to the original draft of the questionnaire following the pilot work. The questionnaire had three sections. The first section of the questionnaire had four questions and was aimed to collect demographic information of the participants including the age, gender, education, and nationality. Section two consisted of one question and was designed to collect the medication history of the participants. Section three comprised of eight questions to explore participants’ practices of fasting during Ramadan, their frequency of medicine intake during fasting, any advice received from their healthcare professionals including doctors and pharmacists, any problems arising from the changes made in their medicine schedule and any fasting considerations for next Ramadan.

Eligible participants (adults aged 18 years or over) diagnosed with diabetes and were attending Diabetes Center of a large governmental hospital in Makkah, Saudi Arabia were included in the study on convenience sampling basis. Patients were excluded if they were non-diabetic or did not provide consent to the study. The quantitative data was entered and coded in Microsoft EXCEL (Microsoft Corporation, Albuquerque, NM, USA). Each question in the questionnaire was considered as categorical variable (nominal or ordinal). The data was mainly presented as frequencies and percentages. Inferential statistics was performed to compare relevant variables/questions using chi-square test. Statistical Package for Social Sciences (SPSS) version 21 (SPSS Inc., Chicago, IL, USA) was used for all data analysis in this phase.

## 3. Results

### 3.1. Qualitative Results

20 patients (10 male, 10 female) attended face-to-face interviews. Two broad categories considered to be possible risk factors for DRPs were developed from participants’ responses: patient-related factors and healthcare professionals-related factors (Table 1). 

### 3.2. Patient-Related Factors

#### 3.2.1. Management of Diabetes during Ramadan

In general, almost all participants observed fasting the whole month of Ramadan. Participants were allowed to fast by their physicians, with exception of two participants who observed fasting without any medical advice. One of the main issues identified by participants was the difficulty in changing the way they took their medicines during the first few days of Ramadan fasting:

“… the medication caused me a nearly strong decline, bringing the level of blood sugar down to 30 or 40” (Patient one).

“… the first 4 days in Ramadan were not easy actually, I do not know if it is because of changing the timing of my medication or because I was fasting, after 4 days things were back to normal …” (Patient two).

“… the way I took my medications wasn’t easy it took some time to settle…” (Patient six).

#### 3.2.2. Diet and Lifestyle during Ramadan

With regards diet and lifestyle during Ramadan, participants had contradicting opinions. Many of the participants thought that their diet regime in Ramadan would help them in controlling their blood glucose: 

“… yes, absolutely diet in Ramadan can replace the need for medications ...” (Patient five).

“… I see Ramadan as an opportunity for meal planning that would help me control my blood glucose …” (Patient nine).

However, a few participants reported that their eating habits in Ramadan had a negative effect on their blood glucose and increased the need for their medications: 

“… the problem is that in Ramadan the meal includes a lot of food and will eat a lot so yes diet in Ramadan increase the need for medications …” (Patient eight).

“… I believe because of my diet habits in Ramadan, there will be more need for the medications.” (Patient 10).

Generally, most of the participants intended to walk during Ramadan with some considering going to mosque every day as an exercise. For some, long hours of working in private jobs in Ramadan was the major reason to not exercise:

“… usually I walk for short distances before iftar, I remember once I did try to exercise more than just waking and I ended up with my glucose level going down. Then I realized I should only walk for short distance …” (Patient one).

“… of course, I cannot run while I’m fasting, but walking is good choice in Ramadan for me, it’s hard to manage the time in Ramadan for the gym but I usually walk only in Ramadan.” (Patient six)

### 3.3. Healthcare Professionals-Related Factors

#### Communication between Healthcare Professionals and Patients

In general, many participants highlighted the lack of effective communication between patients and health care providers—in particular, pharmacists:

“… yes, there is a lack of pharmacist’s role...” (Patient two).

“… my previous doctor was the only one who would care about my medication, he used to talk to me a week before Ramadan start, but I have moved here to another doctor lately and he did not mention anything about Ramadan, not even the pharmacist …” (Patient three).

“… yeah they dispense the medications without instructions …” (Patient seven).

### 3.4. Quantitative Results

95 (40 male, 55 female) completed the questionnaire in the second phase of the study. Majority of the participants (97%) were Saudi nationals with more than half (53%) between 40–59 years. Only 20% had primary education while 26% had no formal education. Well over half (63%) of the participants were taking more than three medicines including medicines for diabetes. Majority of the participants (77%) had fasted the full Ramadan month. However, just over half (51%) reported to have received advice about fasting from healthcare professionals (Table 2). 

Over half of the participants (54%) reported to have made changes to their medicines schedule during Ramadan fasting; however only 15% went on to completely stop taking their medicines during the month of fasting. 41% of the participants who observed fasting reported to have experienced general healthcare problems. The most frequent problems reported by patients included hypoglycemia, hyperglycemia, fatigue, excessive sweating, and gastrointestinal disturbances.

Almost all (95%) of the participants expressed their desire to fast during next Ramadan with full interest and excitement. A significant association (*p* = 0.04) was reported between participants who had not received formal education with the incidence of health-related problems during Ramadan fasting. None of the other variables including age, gender, and number of medicines used were reported to be significantly associated with the incidence of health-related problems.

## 4. Discussion

To the authors’ knowledge, this is the first study that has used both quantitative and qualitative approaches to identify the risk of DRPs in diabetic patients in relation to their practices towards medications intake during Ramadan in Saudi Arabia. The mixed-methods approach used in this study allowed the authors to address the limitations of both qualitative and quantitative methods by conducting exploration as well as analysis in the single study. Thus, the use of combined methodology allowed the authors to develop a more comprehensive and broader perspective of DRPs among diabetic patients. The study has identified two potential risk factors for DRPs: patient-related factors including changes in medicines intake during fasting and healthcare professionals-related factors including lack of advice from healthcare professionals regarding fasting. 

Over half of the participants (54%) were reported to have made changes to their medicines schedule during Ramadan fasting. Furthermore, as evident from the qualitative analysis of the study, many participants reported the challenges and difficulties in adapting to their new medication schedule particularly in the first few days of Ramadan. It is also important to highlight here that just over just over half (51%) reported to have received advice about fasting from healthcare professionals that highlights the lack of effective communication between healthcare professionals and patients. Pharmacists who are expected to provide advice to patients on their medicine intake were not found to be supportive by the participants of the study. Lack of support by pharmacists was attributed to poor communication between patients and pharmacists. Patients reported that pharmacists’ role was limited to supply of medicines only and did not extend to the provision of advice related to the use of medicines or the management of condition. The self-alteration of medicines schedule by patients without receiving advice from healthcare professionals can put the patients at risk of developing potential DRPs. The healthcare professionals including pharmacists should therefore need to actively engage with the patients who are at risk of DRPs by providing Ramadan specific education and counseling.

Lifestyle including diet and exercise during Ramadan was perceived both positively and negatively by participants in the study. Around 80% of the participants believed that change in diet habits in Ramadan would affect diabetes control with some believing that the change was associated with an improvement in their blood glucose. However, for few, irregular eating patterns in Ramadan had a negative impact on their blood glucose and led to an increased reliance on their medications. Similar findings have also been reported in two previous studies [17,18] that suggests that diet management is critical in maintaining optimal glucose control during Ramadan. With regards to exercise in Ramadan, walking was considered the most preferred choice of exercise by participants of the study. Longer working hours and the fear of experiencing hypoglycemia during fasting were some of the reasons for not doing regular exercise by the participants of the study. 

Ramadan fasting in general was perceived very positively by majority of the participants of this study. Almost all (95%) participants indicated their willingness to fast during future Ramadan. The positive perception of participants towards Ramadan fasting and its perceived health benefits has also been reported in two previous qualitative studies conducted in Denmark [19] and Sweden [20]. Patients who choose to fast may be prepared to sacrifice their experience of occasional ill effects (decreased or increased blood glucose) for the religious and socials gains associated with fasting. The healthcare professionals should therefore recognize the spiritual needs of patients when counseling them and not simply advise them against fasting [21]. An open discussion that is receptive to patients’ religious beliefs will enable healthcare professionals to formulate and deliver patient-centered advice related to fasting. Such discussions would also be expected to yield improved clinical outcomes with better compliance to treatment [22]. 

This study has some limitations. No power calculation was undertaken prior to the commencement of this study. However, it may be argued that it was a descriptive study with no hypothesis testing. Participants were included in the study based on their willingness and ability to participate in the study. In other words, the sample population used was a convenience sample of patients. Another limitation was the time period used to collect study data. Data was collected six months after Ramadan that may indicate that participants might have forgotten some of their experiences during last Ramadan. 

Nevertheless, this study has identified several potential risk factors for DRPs that can help healthcare professionals in developing comprehensive educational programs for patients with long-term medical conditions such as diabetes. Patients should be advised to seek detailed advice from their healthcare professionals about the benefits and risks associated with fasting. Patients who insist on fasting should be provided explicit education about the schedule of their medicine intake during fasting, self-monitoring of blood glucose levels, recognition of signs and symptoms of low or high blood glucose levels and importance of undertaking regular physical activity to minimize the incidence of general health or DRPs. The findings of this study have implications for healthcare professionals and relevant stakeholders in other neighboring Gulf countries due to similarities in patients’ demographics and practices during Ramadan. 

## 5. Conclusions

Diabetic patients who fast during Ramadan may alter their medicines schedule without seeking medical advice from healthcare professionals. Healthcare professionals need to educate such patients by providing structured education and counseling to avoid the risk of developing DRPs.

## Figures and Tables

**Table 1:** Themes and subthemes generated from participants’ responses.

Category	Themes and Subthemes Identified within this Category
Patient-related factors	Management of diabetes during Ramadan ➢ Knowledge about medicines ➢ Number of medicines taken ➢ Frequency of medicine intake ➢ Medicine adherence ➢ Changes in medication intake ➢ Adaptability to changed medicine schedule ➢ Over dosage ➢ Stopping medicine intake during fasting ➢ Health problems related to medicines use ➢ Recognizing the signs of high or low blood glucose ➢ Monitoring blood glucose
	Diet ➢ Change in dietary habits ➢ Irregular eating ➢ Overeating ➢ Lack of healthy diet
	Lifestyle ➢ Type of exercise during fasting ➢ Lack of time for exercise ➢ Walking ➢ Longer working hours ➢ Duration of exercise ➢ Smoking ➢ Irregular sleeping patterns
Healthcare professionals-related factors	Communication between healthcare professionals and patients ➢ Barriers to communication ➢ Doctors busy schedule ➢ Lack of advice from pharmacists
	Future considerations for fasting ➢ Seeking permission from doctors before fasting
	Seeking advice before making changes in medicine schedule

**Table 2:** Quantitative description of the participants’ responses to the questionnaire.

Quantitative Description of the Participants’ Responses to the Questionnaire			
Gender	male	40	42%
	female	55	58%
Age	Less than 20 years	4	4.20%
	20–39years	9	9.50%
	40–59 years	50	52.60%
	60 years or above	32	33.60%
Education	No formal education	25	26.30%
	Primary school	19	20%
	Secondary school	24	25.20%
	College/university	27	28.40%
Nationality	Saudi	92	97%
	Non-Saudi	3	3%
How many medicines do you currently take in total?	Less than 3 medicines	20	21%
	Three medicines	14	15%
	More than 3 medicines	60	63.10%
	4	1	2.50%
Has the doctor/nurse/pharmacist ever explained to you what to do with your medicines (i.e., how to take them (doses), how many times and when] in Ramadan?)	Yes	88	92.60%
	No	7	7.30%
How many days did you fast?	Less than half of the month	9	9%
	More than half of the month	13	14%
	Full month	73	77%
Did your doctor advise you about fasting in Ramadan?	Yes, my doctor advised me that I can fast in Ramadan	49	51%
	No, my doctor advised that I cannot fast in Ramadan	4	4.20%
	My doctor did not advise anything about fasting in Ramadan	41	43%
Did you feel any general health problems such as low or high blood glucose, fatigue, and gastrointestinal disturbances during fasting?	Yes	39	41%
	No	56	59%
Did you change the way (e.g., increased or decreased the dose) of your medicines in Ramadan?	Yes	52	54%
	No	43	45%
Have you ever stopped taking any medicine at all while fasting in Ramadan?	Yes	14	15%
	No	81	85%
Do you think changing the eating habits in Ramadan (whether fasting or not) affected your diabetes control?	Yes	76	80%
	No	19	20%
Will you consider fasting next Ramadan?	Yes	90	95%
	Not sure	5	5%

## Appendix A: Interview Guide

### Start/Introduction

“Salam Alaikum. My name is ... and I am a pharmacy student from Umm Al-Qura University. We are doing a research in which we are asking people with diabetes if they face any problems related to their medicines while fasting in Ramadan. Is it OK with you if I ask you some questions about this?”

### Proceed if the patient shows willingness.

“Thank you for accepting to take part in our research. I will ask you some questions in this short interview about your medicines and fasting in Ramadan. We hope we can use this information to understand the problems better and help other diabetic patients in future.

Before we start, I would just like to remind you of a few things:

1. The information you will provide will remain completely anonymous and confidential. I will audio record your interview, so we can analyze this information later to know about the general problems.
2. If at any time during the interview, you do not feel comfortable/good or would not like to answer a question, please let me know—we will skip the question and move on.
3. If you feel like not satisfied with any of your answer, please inform me, and we will delete that answer from the recording.

Shall we continue?”

### Press the Record Button & Start with Saying Patient Number Which + Your Name.

#### 1. Medicines

1. Can you please tell me the name of medicines which you take for diabetes and how many times you take them?
2. Has any of your diabetes medicines changed since the time you were diagnosed with diabetes?
3. Has the doctor/nurse/pharmacist ever explained to you what to do with your medicines (i.e., how to take them, how many times and when) in Ramadan?

#### 2. Ramadan

1. Did you fast during last Ramadan or have you ever fasted in Ramadan?

For Patient who Has ever Fasted with Diabetes in Ramadan

2. How many days did you fast?
  If not all Days: can you please tell me why you left some of the fasts during Ramadan? Is it because of diabetes or medicines ...? Probe the patient to take more detailed views.
3. Did your doctor advise you to fast OR say that you can fast in Ramadan?
  If Yes: Why do you think your doctor advised you that you can fast in Ramadan (e.g., is your health good OR diabetes controlled)?
  If No: Can I ask you why you did not act on doctor’s advice?
4. Did you feel any problem with your general health or diabetes when you fasted in Ramadan?
  If Yes: Can I ask you what problems? Probe the patient to know if it could be related to medicines.
5. Did you change (e.g., increase or decrease the medicines or miss some medicines) the way you take your medicines in Ramadan as compared to the normal days outside Ramadan?
  Refer to Q3 and probe the patient about each medicine.
  If No: Can you please tell me how you were taking your medicines in Ramadan i.e., how many times? Ask about each medicine and compare it with info provided in Q3 for verification.
  If Yes: Was this advised by the doctor/nurse/pharmacist?
  If No: How did you decide how to change the way you should take your medicines in Ramadan?
6. Was it easy to change the way of taking medicines in Ramadan?
  Probe the patient to take more detailed views.
7. Did you feel/face any problem because of changing the way of taking medicines in Ramadan?
  If Yes: Did you inform the doctor or pharmacist about this?
  If No: Why?
8. Did you stop/have you ever stopped taking any medicine at all while fasting in Ramadan?
  If Yes: Which one? Why?
9. Did you ever break/have you ever broken your fast because of diabetes or because you wanted to take the medicine in Ramadan?
  If Yes: Which medicine you wanted to take?
10. Do you measure your blood sugar level during fasting?
  If No: Why?
  If Yes: How many times and how?

For Patient who has Never Fasted with Diabetes in Ramadan

2. Did your doctor advise you not to fast OR say that you cannot fast in Ramadan?
  If Yes: Why do you think your doctor advised you that you cannot fast (e.g., is it because of your general health or diabetes)?
  If No: Can I ask you why you decided not to fast during Ramadan (e.g., is it because of diabetes or medicines …?
  Probe the patient to take more detailed views.
3. If you fast in Ramadan, do you think your general health or diabetes can be affected?
  If Yes: how?
4. If you fast in Ramadan, do you think you would not be able to take the medicines in the way you take normally outside Ramadan?
  If Yes: How do you think it (changing the way medicines are taken) would affect your diabetes control?
5. Do you monitor your blood sugar level?
  If Yes: How many times and how?
  - If you fast in Ramadan, do you think you would not be able to monitor your blood sugar level while fasting?
  If Yes: Why?
6. Will you try and fast next Ramadan?

#### 3. Exercise

1. Do you exercise/walk (generally outside Ramadan and in Ramadan)?
  If No: Can I ask you why (e.g., you are busy OR it is not easy for you)?
  If Yes:
  - What exercise do you do OR how much do you walk?
  - Does this help to control your diabetes?
  - Do you think fasting in Ramadan can affect your exercise/walk?
  Probe the patient to take more detailed views.

#### 4. Dietary Habits

1. How do you think change in eating habits in Ramadan affect your diabetes control?
2. Can it (different dietary habits in Ramadan) have any effect on the medicines you take (e.g., you take more or fewer medicines or miss some medicines)?
  If Yes: How?

#### 5. Other Conditions & Medicines

1. Do you have any other diseases or problems in addition to diabetes?
  If Yes: What are they?

#### 6. Disease/Medicines Management

1. Who helps you take your medicines at home?
2. Does your family support or help you control your diabetes?
  If Yes: How?
3. How do you know/feel when your diabetes is controlled?
4. How do you know/feel when your diabetes is not controlled?
  - What do you do in that situation (and why)?

#### 7. Future

1. Will you try and fast next Ramadan?
2. Do you have any suggestions for doctor/nurse/pharmacist how they can help you to better control your diabetes and take medicines in Ramadan next year?

End/Close

“This is the end of interview. Thank you very much for your time.”
